# Identification, Characterization and Expression Profiles of *Xylogen-like* Gene Family in Kiwifruit in Different Developmental Tissues and Under Various Abiotic Stresses

**DOI:** 10.3390/biology15030264

**Published:** 2026-01-31

**Authors:** Caili Wang, Chen Li, Xiaoli Tang, Chunhua Li

**Affiliations:** 1 College of Life Sciences, Dezhou University, 566 University Road, Dezhou 253023, China; 2 The Engineering Research Institute of Agriculture and Forestry, Ludong University, 186 Hongqizhong Road, Yantai 264025, China

**Keywords:** *XYLP* gene family, abiotic stress, expression profiles, kiwifruit

## Abstract

The *XYLP* family is reported to be essential for the regulation of growth and development, mediating plant hormone signals, and promoting adaptation to environmental stresses across various plant species. Although the *XYLP* family in *Actinidia chinensis* (kiwifruit) enhances the understanding of developmental processes and abiotic stress responses, a systematic analysis of this gene family remains lacking. In this study, 28 *AcXYLP* genes were identified in the kiwifruit genome, which were classified into five clades based on a phylogenetic tree. Collinearity analysis revealed that the expansion of *AcXYLP* genes was predominantly influenced by segmental genome duplication events. Gene ontology and protein–protein interaction network analysis indicated that *AcXYLP* genes were involved in pathways related to plant growth, developmental process, and abiotic stresses responses. The promoter regions harboring *cis*-acting elements associated with plant growth and development, phytohormones, and stress responses, highlighting the significant roles of the *AcXYLP* gene family. Expression profile analysis of 28 *AcXYLP* genes in different tissues revealed their tissue-specific functions. Notably, *AcXYLP13*, *AcXYLP15* and *AcXYLP27* were identified as top-ranked nodes in STRING-predicted network protein involved in plant growth and abiotic stress responses, indicating their central roles in coordinating developmental signaling and stress responses. This study facilitated further exploration of the functional characteristics of *AcXYLP* genes.

## Simple Summary

This study investigates the *XYLP* gene family in kiwifruit, focusing on its roles in growth, development, phytohormone response, and abiotic stress. A total of 28 *AcXYLP* gene family members were identified and categorized into five clades. Most AcXYLPs are alkaline, predominantly hydrophobic, and tend to be structurally unstable. All the AcXYLPs are in silico predicted to be localized to the cell membrane. The 28 *AcXYLP* genes exhibit uneven distribution, with segmental duplication as the major driving force during gene family expansion. Transcriptome analysis, complemented by qRT-PCR validation, confirmed the involvement of *XYLP* genes in organ development, fruit ripening, and responses to phytohormones and abiotic stresses. This study lays a foundation for future investigations into the biological functions of *XYLP* genes in kiwifruit.

## 1. Introduction

In higher plants, many extracellular proteins are involved in various biological processes, including intercellular communication, growth and developmental processes, as well as stress responses. Arabinogalactan proteins (AGPs), as extracellular proteins, are widely distributed in the plant kingdom and algae species, and are among the most complex macromolecule families in plants [1,2]. AGPs are a class of glycoproteins composed of large type II arabinogalactan (AG) glycan chains and a protein backbone rich in proline/hydroxyproline (P), alanine (A), serine (S), and threonine (T) [3,4,5,6]. The AG glycan chain and the protein backbone are linked together through O-glycosylation of hydroxyproline [6]. AGPs are present in almost all plant organs and cell types, and are involved in multiple processes such as cell expansion [7,8], cell proliferation [9], embryonic development [10,11], pollen tube growth [12], pollen development [13], cell wall remodeling [14], xylem differentiation [15], root development [16], responses to brassinosteroids [17] and salt stress [18].

The high functional diversity of AGPs can be attributed to the high complexity and specificity of their carbohydrate moieties and protein backbone composition [2,3]. In addition to the AGP domain, chimeric AGPs also possess other conserved domains. Based on the composition of conserved domains, chimeric AGPs can be further classified into plastocyanin-like AGPs (PLAs) [19,20], fasciclin-like AGPs (FLAs) [21], and xylogen-like proteins (XYLPs) [22]. As chimeric AGPs, the XYLPs have a unique structure containing an AGP domain and a conserved non-specific lipid-transfer protein (nsLTP) domain [23]. nsLTPs are a class of small, soluble and basic proteins belonging to a complex, multigene family, primarily defined for their ability to transport lipid molecules in vitro [24]. The nsLTP exhibits high stability, which is attributed to its unique structure: it contains eight conserved cysteine residues that form four disulfide bonds, thereby stabilizing the protein’s tertiary structure [25]. Xylogen is first isolated and identified as an intercellular signaling molecule from suspension-cultured *Zinnia* leaf cells [26,27]. Previous studies have shown that xylogen, secreted by differentiating vascular cells, can be transported to adjacent undifferentiated cells and promote their differentiation into tracheary elements [22].

The fine regulation of vascular differentiation is triggered by various signaling regulators, including phytohormones (such as auxin, cytokinin and brassinolide), xylogen and plant small peptides, among which xylogen plays a crucial role [28]. In *Arabidopsis*, the double knockouts of *xyp1 xyp2* exhibit obvious defects in vascular structure, with discontinuous veins and vessels in leaves and roots [22]. The *xylp7* T-DNA insertion mutant in rice demonstrates a shorter length of internodes except for the basal internode [29]. The overexpression of *PtXYLP1* results in the defective venation pattern in *Arabidopsis* cotyledons [30]. Meanwhile, there may be a close interaction between phytohormones and xylogen, as the expression of xylogen is upregulated by auxin, cytokinin and gibberellin. In addition to plant growth, xylogen may also be involved in plants’ response to abiotic stress, with *XYLP* genes being almost regulated by salt, drought and cold treatment [29,31].

The identification at a genome-wide scale of the xylogen-encoding gene family has been achieved as an effective strategy for gene function analysis in species such as *Arabidopsis* [23], rice [29], poplar [30], and moso bamboo [31]. However, no relevant studies on members of XYLPs have been reported in kiwifruit or other wine plants to date. As an economically important fruit crop, kiwifruit is rich in vitamins and minerals, yet the cultivation is often challenged by abiotic stresses, resulting in reduced yield [32,33]. Prompted by these unanswered questions, we performed a comprehensive bioinformatics analysis of the *XYLP* gene family in the kiwifruit genome. In *Actinidia chinensis*, 28 *AcXYLP* genes in total were identified. The physicochemical properties, phylogenetic relationships, gene and protein structures, chromosomal locations, duplication events, gene ontology (GO) annotations, protein–protein interaction networks, and promoter *cis*-acting elements were systematically characterized. The expression patterns of seven representative *XYLP* genes were also investigated in response to various abiotic stresses and phytohormone treatments. The results presented here serve as a resource for the *XYLP* family in kiwifruit and will facilitate targeted functional investigations of its members for further studies.

## 2. Materials and Methods

### 2.1. Sequence Identification and Characterization

Protein sequences of 13 AtXYLP were retrieved from The Arabidopsis Information Resource (https://www.arabidopsis.org/). Those sequences served as queries for a BLASTP (v2.13.0+) search against the Kiwifruit Genome Database 3.0 (https://kiwifruitgenome.atcgn.com) [34] with an *E*-value threshold < 1 × 10^−7^. Iterative BLASTP searches were performed using newly identified candidates as queries until no further homologs were found. Sequences were identified as AcXYLPs [29] based on the presence of both predicted arabinogalactan (AG) glycomodules, identified manually according to established criteria, by analyzing the presence of the Pro/Ala/Ser/Thr (PAST)-rich motifs as AGP markers in the sequence which contained at least one region with ≥35% PAST residues [30,35], and an nsLTP-like domain (PF00234). Domain prediction was performed via the Pfam hidden Markov model (HMM) (http://pfam.xfam.org/) and subsequently validated using InterProScan 108.0 (https://www.ebi.ac.uk/interpro/, accessed on 29 January 2026) [36] with default settings. For comparative analysis, 31 poplar XYLP (PtXYLP) sequences were obtained from Phytozome 14 (https://phytozome-next.jgi.doe.gov/), as described previously [30].

### 2.2. Analysis of Physicochemical Properties and Protein Structure

The physicochemical properties, including theoretical isoelectric point (pI) and molecular weight (MW) of AcXYLPs were predicted using the ExPASy ProtParam tool (https://web.expasy.org/protparam/, accessed on 29 January 2026) [37]. The generation of the hydropathicity profiles was applied with ProtScale (https://web.expasy.org/protscale/, accessed on 29 January 2026) [37], using the Kyte–Doolittle scale with a window size of 9. SignalP 6.0 (https://services.healthtech.dtu.dk/services/SignalP-6.0/, accessed on 29 January 2026) was introduced in the prediction of the signal peptides of the AcXYLPs [38] with the default organism setting “Eukarya”. Potential GPI-anchor modification signals were predicted using NetGPI 1.1 (https://services.healthtech.dtu.dk/services/NetGPI-1.1/, accessed on 29 January 2026) [39]. The online tool NetNGlyc 1.0 (https://services.healthtech.dtu.dk/services/NetNGlyc-1.0/, accessed on 29 January 2026) [40] was used in the N-linked glycosylation site prediction, applying a potential threshold of >0.5. Secondary structure composition (alpha-helix, extended strand, random coil) was predicted using the SOPMA method via the NPS@ server (https://npsa-prabi.ibcp.fr/cgi-bin/npsa_automat.pl?page=/NPSA/npsa_sopma_f.html, accessed on 29 January 2026) [41] by using the following parameters: similarity threshold = 8, window width = 17, and the default parameters of the tool. Tertiary structures were modeled using the automated mode of SWISS-MODEL (http://swissmodel.expasy.org/) [42], and model quality was assessed based on GMQE scores.

### 2.3. Phylogenetic, Genomic, and Gene Structure Analysis

Multiple sequence alignment of AtXYLP, AcXYLP, and PtXYLP was conducted with the built-in ClustalW tool using default parameters using MEGA 11. Subsequently, a phylogenetic tree was constructed from this alignment using the neighbor-joining (NJ) method in MEGA 11 [43,44], applying the Poisson model, pairwise deletion, and 1000 bootstrap replicates. Chromosomal locations, segmental duplication events, and genomic collinearity among *A. thaliana*, *A. chinensis*, and *P. trichocarpa* were analyzed using MCScanX v1.0.0 [45] with the following key parameters: match score = 50, match size = 5, e-value = 1 × 10^−10^, max gaps = 25. The resulting collinear blocks were visualized using the “Dual Synteny Plot” function in TBtools v2.311 [46]. The ratios of non-synonymous (Ka) to synonymous (Ks) substitutions for duplicated gene pairs were calculated with the “Simple Ka/Ks Calculator” in TBtools [46]. Gene structures (exon-intron organization) were visualized with the Gene Structure Display Server 2.0 (http://gsds.cbi.pku.edu.cn/) [47] using genomic and CDS sequences as input. Conserved motifs within the nsLTP domain were aligned using MUSCLE (v3.8.31, https://www.ebi.ac.uk/jdispatcher/, accessed on 29 January 2026) [48] with default settings and rendered as a sequence logo with WebLogo 3 (https://weblogo.threeplusone.com/) [49].

### 2.4. Promoter and Functional Annotation Analysis

Sequences spanning 2000 bp upstream of the translational start site of each *AcXYLP* gene were retrieved and screened for cis-acting elements via the PlantCARE database (http://bioinformatics.psb.ugent.be/webtools/plantcare/html/, accessed on 29 January 2026) [50] with default settings. Gene ontology (GO) annotation was performed by submitting AcXYLP sequences, using the *Arabidopsis thaliana* GO information as a reference, with the “Go Annotation” module built in TBtools. Enrichment analysis results were visualized using the “ggplot2” module within TBtools [46]. Protein–protein interaction (PPI) networks were predicted using the STRING database (v12.0, https://cn.string-db.org/) with the following parameters: organism “*Actinidia chinensis*”, meaning of network edges set to “confidence”, and a minimum required interaction score of 0.400 (medium confidence). Functional enrichment in the network was ranked based on the “node degree” parameter, as provided in the “string_node_degrees.tsv” file downloaded from the STRING Exports page. The resulting network was downloaded and visualized using Cytoscape (v3.10.4, https://cytoscape.org/).

### 2.5. Plant Materials and Treatments

Seeds of *A. chinensis* “Hong Yang” were surface-sterilized and germinated on half-strength Murashige and Skoog (MS) medium supplemented with 0.8% agar at 24 °C under a 12 h light (8000 lux)/12 h dark photoperiod. For tissue-specific expression analysis, samples from shoot apices (AP), young leaves (YL, the 1st and 2nd leaves from the apex), mature leaves (ML, fully expanded leaves), young stems (YS, internodes of freshly growth), mature stems (MS, lignified stems), and roots (RT) were collected from 3-month-old soil-grown plants. For phytohormone treatments, the aerial parts of 4-week-old seedlings were evenly sprayed with 100 μM solutions of abscisic acid (ABA), salicylic acid (SA), or methyl jasmonate (MeJA), and collected at 3 and 12 hours post-treatment. For abiotic stress treatments, 4-week-old plants were subjected to (i) low temperature (4 °C in a growth chamber), (ii) drought stress (supplied with 10% (*w*/*v*) PEG6000 solution), or (iii) salt stress (supplied with 200 mM NaCl solution), and collected at 6 and 12 hours post-treatment. Upon collection, all tissue samples were immediately frozen in liquid nitrogen and subsequently maintained at −80 °C. For each treatment, three biological replicates were established, each constituted by pooling tissues from a minimum of five plants.

### 2.6. Expression Analysis

The in silico expression analysis was performed on a laboratory server running Ubuntu 22.04.3. Expression profiles were derived from publicly available RNA-seq datasets in the NCBI SRA database, specifically under accessions PRJNA328414 (various tissues) and PRJNA187369 (fruit development stages: DAP20, DAP120, DAP127). The NCBI SRA Toolkit (https://github.com/ncbi/sra-tools/wiki/01.-Downloading-SRA-Toolkit, accessed on 29 January 2026) was used to retrieve sequencing data. SRA records listed in index.txt were downloaded using the command prefetch --option-file ./index.txt. FASTQ files were then extracted from the downloaded SRA files with the command fastq-dump --split-3 ./SRR.sra. Raw sequencing reads were pseudoaligned and quantified using Kallisto (v0.50.0) [51] to obtain Transcripts Per Million (TPM) values. A kiwifruit transcriptome index was first constructed with the command kallisto index ./Ac.cds.fa -i ./index. Quantification was then performed using kallisto quant -i ./index -o output_dir -b 100 ./SRR_1.fastq ./SRR_2.fastq. The “Heatmap” module in TBtools [46] was employed to generate clustered heatmaps with the following parameters: clustering method = “Average” and distance metric = “Euclidean”.

For the validation of the expression profile, total RNA was extracted using the Polysaccharide Polyphenol Plant Total RNA Kit (Tiangen, Beijing, China, DP441) following the manufacturer’s protocol, including an on-column DNase I digestion step. RNA concentration and purity were assessed by NanoDrop (Wilmington, DE, USA) and agarose gel electrophoresis. For cDNA synthesis, 1 µg of total RNA was used in the reverse-transcription, by using the PrimeScript™ RT Master Mix (Takara, Beijing, China, RR036A) according to the manufacturer’s instructions, in a final volume of 20 µL. Gene-specific primers used in quantitative real-time (qRT)-PCR are listed in Table S1. qRT-PCR analyses were conducted on an Agilent Mx3000P system (Agilent Technologies, Santa Clara, CA, USA), employing TB Green^®^ Premix Ex Taq™ II (Takara, RR820A), in a final volume of 20 µL. The thermal cycling conditions were: 95 °C for 30 s, followed by 40 cycles of 95 °C for 5 s, 58 °C for 30 s, and 72 °C for 15 s, with a subsequent melting curve analysis (95 °C for 15 s, 60 °C for 60 s, 95 °C for 15 s) to confirm primer specificity. For normalization purposes, *AcActin* was used as the reference gene, and relative expression was calculated via the 2^−ΔΔCt^ method. All the samples and qRT-PCR reactions were run in triplicate, and each sample consists of 3–5 kiwifruit plants.

### 2.7. Subcellular Localization

Subcellular localization was predicted in silico using Plant-mPLoc (http://www.csbio.sjtu.edu.cn/bioinf/plant-multi/, accessed on 29 January 2026) [52]. For experimental validation, the full-length coding sequences of *AcXYLP1* and *AcXYLP27* (without the stop codon) were amplified and cloned into the pSAT6-EYFP-N1 vector (via restriction enzymes *Bam*HI and *Spe*I) to generate C-terminal fusions with GFP under the control of a double CaMV 35S promoter. The recombinant plasmids (*35Spro::AcXYLP*-*GFP*) and control vectors (*35Spro::GFP*) were purified using a Plasmid Miniprep Kit (Axygen, Union City, CA, USA). Arabidopsis leaf mesophyll protoplasts were isolated from 4-week-old Col-0 plants using an enzyme solution (1.5% cellulase R10, 0.4% macerozyme R10, 0.4 M mannitol, 20 mM KCl, 20 mM MES pH 5.7, 10 mM CaCl_2_, 0.1% BSA). Approximately 2 × 10^4^ protoplasts were transfected with 20 µg plasmid DNA via the PEG-calcium method [53]. For co-localization studies, protoplasts were co-transfected with the GFP-fusion constructs and the plasma membrane marker SCAMP1-RFP [54] or the tonoplast marker TPK1-RFP [55] at a 1:1 molar ratio. After incubation in the dark at 22 °C for 16–20 h, fluorescence signals were observed using a confocal laser scanning microscope (Zeiss LSM 880, Jena, Germany) with excitation/emission settings of 488 nm/505–530 nm for GFP and 561 nm/575–620 nm for RFP. Co-localization was assessed using the Pearson correlation coefficient. For each condition, images were acquired from 5 to 10 cells per field of view, and quantification was performed on 20–30 cells per replicate. All experiments were independently repeated at least three times.

### 2.8. Statistical Analysis

Data were presented as the mean ± SD from a minimum of three independent biological replicates, with all statistical analyses and graphs generated using GraphPad Prism software (v 10.6). For qRT-PCR-derived tissue-specific expression profiles, statistical significance was determined by one-way ANOVA with Tukey’s HSD post hoc test for pairwise comparisons against controls at each time point; significance was denoted as *p* < 0.05 (*), *p* < 0.01 (**), and *p* < 0.001 (***). For the expression profile analyses under phytohormone and abiotic stress treatments, a two-way ANOVA was conducted, and where overall effects were significant, pairwise comparisons were further examined using Tukey’s HSD test, with statistically significant differences (*p* < 0.05) indicated by different superscript letters in the corresponding figure.

## 3. Results

### 3.1. Identification, Physicochemical Properties, and Evolutionary Relationship of the AcXYLP Family Members

**Figure 1:**
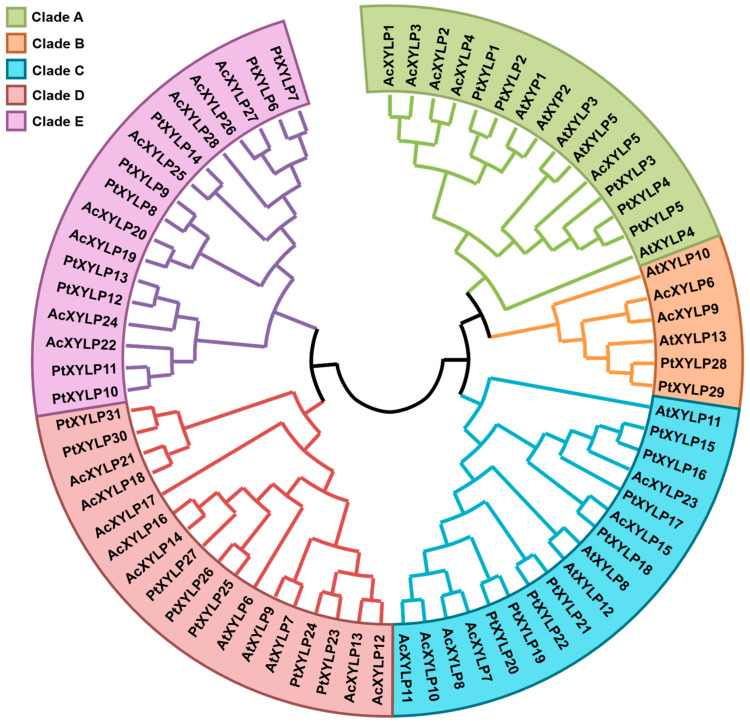
Phylogenetic relationships of XYLPs from kiwifruit, *Arabidopsis* and poplar. The phylogenetic tree was constructed by the neighbor-joining method using MEGA 11, with 1000 bootstrap replicates. The abbreviations “*Ac*” for *Actinidia chinensis*, “*At*” for *Arabidopsis thaliana*, “*Pt*” for *Populus trichocarpa* were placed before the gene family name (*XYLP*).

To identify putative *XYLP* gene family members in kiwifruit, a BLASTP search was conducted against the kiwifruit genome database, with the protein sequences of ZeXYP1 and AtXYLPs used as queries, followed by a self-BLASTP search with the candidate kiwifruit XYLPs [22]. The potential XYLP members were verified for the presence of AGP and conserved nsLTP domain, ultimately identifying a total of 28 *XYLP* genes in the *Actinidia chinensis* genome, which share significant sequence similarity (*E*-value < 1 × 10^−7^). For convenience, the abbreviation “*Ac*” for *Actinidia chinensis* was placed before the gene family name (XYLP). Based on the BLASTP results, they were named as AcXYLP1-AcXYLP28, and the basic AcXYLP physicochemical properties are shown in Table 1. A phylogenetic tree was generated from 72 XYLPs from *Arabidopsis* (dicotyledonous herb model plant), poplar (dicotyledonous woody model plant) and kiwifruit. In the unrooted phylogenetic tree, AcXYLPs were classified into five clades (Clade A–E). Further analysis of the phylogenetic tree revealed Clade E comprised poplar and kiwifruit XYLPs. The absence of *Arabidopsis* corresponding genes suggests that this branch may evolved independently in woody plants (Figure 1).

**Figure 2:**
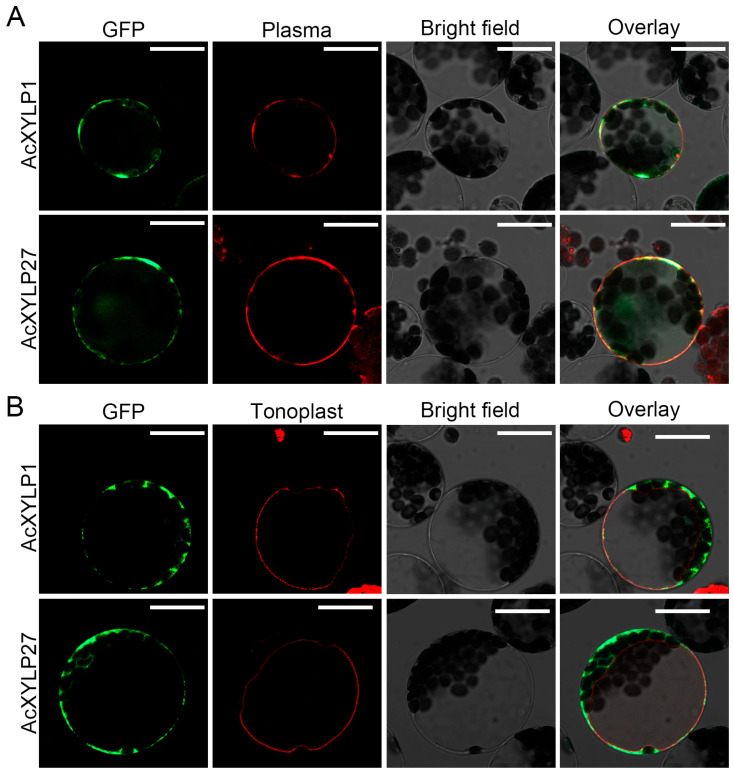
The subcellular localization of the AcXYLPs. Subcellular localization of transiently expressed Green Fluorescent Protein (GFP)-fused AcXYLPs in *Arabidopsis* leaf protoplasts, and observed with laser scanning confocal microscopy. (**A**) The plasma membrane marker Secretory Carrier Membrane Protein (SCAMP)1-Red Fluorescent Protein (RFP) and (**B**) tonoplast marker Two Pore K^+^ (TPK)1-RFP were used to confirm the subcellular localization. Bars = 20 μm. Images were acquired from 5 to 10 cells per field of view, and quantification was performed on 20–30 cells per replicate. A Pearson correlation coefficient (r) threshold of ≥0.85 was applied to ensure the reliability of both image capture and quantitative analysis. All experiments were independently repeated at least three times.

The physicochemical property analysis of the AcXYLPs revealed that the amino acid lengths of the identified proteins range from 143 (AcXYLP26) to 210 (AcXYLP9), with the least (AcXYLP26, 14.90 kDa) and maximum (AcXYLP9, 21.87 kDa) molecular weights observed among the AcXYLPs. The theoretical pI of AcXYLPs ranges from 3.79 to 9.11, with half of the proteins being alkaline (pI > 7.0). The hydropathicity AcXYLPs were mainly positive (except *AcXYLP14*), indicating that they were dominantly hydrophobic. The instability index analysis found that most AcXYLPs were structurally unstable (instability index > 40), except AcXYLP16 and AcXYLP18 (instability index < 40). The prediction of subcellular localization indicated that all AcXYLPs were located on the cell membrane (Table 1). To verify the in silico prediction of AcXYLP subcellular localizations, AcXYLP1 and AcXYLP27 were randomly selected and transiently expressed in *Arabidopsis* mesophyll protoplasts, using SCAMP1-RFP [54] and TPK1-RFP [55] as markers for plasma membrane and tonoplast, respectively. The recombinant vectors of *35Spro::AcXYLP1-GFP* and *35Spro::AcXYLP27-GFP* were constructed and transiently transformed into *Arabidopsis* mesophyll protoplasts via PEG-calcium transfection, respectively. As predicted, the signals of AcXYLP1-GFP and AcXYLP27-GFP fusion proteins were clearly observed in the plasma membrane of *Arabidopsis* mesophyll protoplasts, and no GFP fluorescence signal was present in the tonoplasts (Figure 2).

### 3.2. Gene Structure, Conserved Motif and Protein Structure of the AcXYLP Family Members

**Figure 3:**
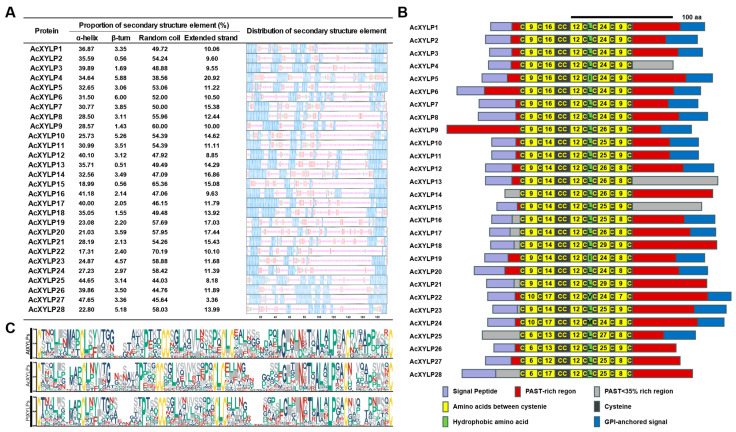
Secondary protein structures and conserved motifs of the AcXYLPs. (**A**) Secondary protein structures of the AcXYLPs. (**B**) Schematic diagram of the AcXYLP structures. (**C**) Conserved nsLTP motifs from *Arabidopsis*, kiwifruit, and poplar.

To further understand the evolutionary relationship of *XYLP* gene family in kiwifruit, the gene structures of *Arabidopsis*, poplar and kiwifruit were analyzed with the Gene Structure Display Server, displayed in Figure S1. Most *AcXYLP* genes contain two introns and three exons, and homologous genes within the same clade exhibit similar exon–intron structures (Figure S1). In Clade E, the *AcXYLP24* gene, along with its homologous genes *PtXYLP10* and *PtXYLP13*, contains three introns and four exons. Notably, the *AcXYLP4* and *AcXYLP25* genes were exceptions, as they lack introns and were composed of a single exon (Figure S1). Secondary-structure analysis was performed on the AcXYLPs, and the results showed that all proteins comprised an α-helix, β-turn, random coil and extended strand, with various proportions and orders of these elements. Among all the AcXYLPs, the random coil occupies the highest proportion, ranging from 38.56% to 70.19%, followed by the α-helix and extended strand. The β-turn occupies the lowest proportion, ranging from 0.56% to 6.0% (Figure 3A). Among the 28 AcXYLPs, 25 were predicted to contain an N-terminal signal peptide, indicating that they could be classified into the secretory pathway as extracellular proteins. Most AcXYLPs were found to have a GPI (glycosylphosphatidylinositol) anchor at the C-terminus, which is a crucial domain for anchoring proteins to the plasma membrane (Figure 3B, Table S2). This result was highly consistent with the subcellular prediction of AcXYLPs (Table 1).

Similarly to other XYLPs, the AcXYLP also contains an nsLTP domain with eight well-conserved cysteine residues arranged in the order of C_1_-X-C_2_-X-P-X-C_3_C_4_-X-C_5_XC_6_-X-C_7_-X-C_8_ (Figure 3C). The hydrophobic residue between C_5_ and C_6_ is typically leucine (L), with only a small proportion of exceptions being isoleucine (I), valine (V), alanine (A), phenylalanine (F) and methionine (M) (Figure 3C). The hydrophobic residues and the conserved eight cysteine residues form a hydrophobic core in the tertiary structure, which is crucial for the lipid binding and transport processes. The glycosylation modification of XYLP is crucial for its catalytic function. All AcXYLPs contain at least one region rich in Pro/Ala/Ser/Thr (PAST) (PAST residues ≥ 35%), which provides moieties for potential glycosylation modifications. Additionally, there was at least one putative AG glycomodule within the PAST-rich region, where the amino acids were regularly arranged as Ala-Pro, Pro-Ala, Ser-Pro, Pro-Ser, Thr-Pro, and Pro-Thr (Table S2). The tertiary structures of the AcXYLPs were further predicted by SWISS-MODEL, with significant differences observed in the aggregation angle and structural adjacency of amino acids among each AcXYLP, which may be attributed to variations in the arrangement and proportion of secondary structural elements within the proteins (Figure 3A and Figure S2, Table S9). As expected, the high sequence similarity and tertiary structure of the nsLTP domain indicate its functional conservation (Figure S3, Table S10). Therefore, the analysis of gene structures, conserved motifs, and protein structures indicated high evolutionary conservation within the *XYLP* gene family.

### 3.3. Chromosomal Localization, Duplication and Collinear Relationship of the AcXYLP Family Members

**Figure 4:**
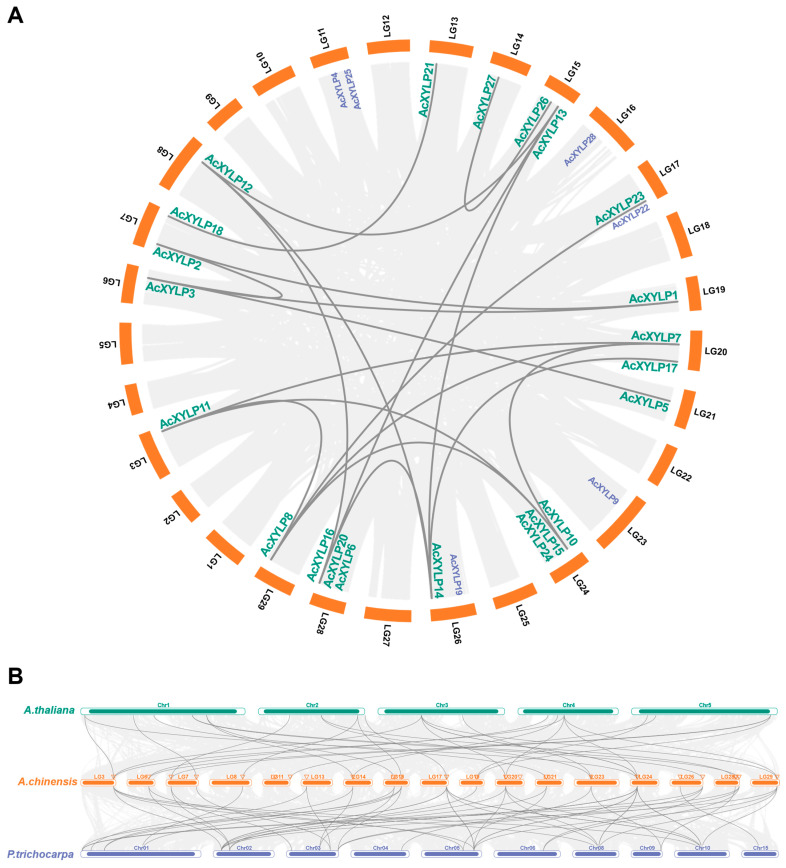
Chromosomal distribution and synteny analysis of *XYLP* genes in kiwifruit, *Arabidopsis*, and poplar. Gray lines showed collinear gene pairs. (**A**) Kiwifruit chromosomes (LG1 to LG29) are represented by orange boxes of varying lengths. (**B**) Illustration of synteny relationships between different XYLP genes.

The 28 *AcXYLP* genes were unevenly distributed on the 18 kiwifruit chromosomes. Specifically, there were three genes on chromosomes 24 and 28; two genes on chromosomes 7, 11, 15, 17, 20 and 26; and the remaining 10 genes were uniformly distributed, with one gene on chromosomes 3, 6, 8, 13, 14, 16, 19, 21, 23 and 29 (Figure 4A). As a fundamental evolutionary mechanism, gene duplication is common to all species and plays a pivotal role in the generation of new genes and species evolution [56]. The result showed that the *AcXYLP* genes had undergone 19 segmental duplication events, with no tandem duplication events observed in the kiwifruit genome, indicating that segmental duplication events might be the major force in the expansion of the *AcXYLP* gene family (Figure 4A). Furthermore, Ka/Ks ratios were calculated to evaluate the evolutionary selection pressure acting on the *AcXYLP* gene pairs generated by segmental duplication events. Among the 19 *AcXYLP* pairs analyzed, 18 exhibited Ka/Ks ratios below one. The pair *AcXYLP14/AcXYLP17* was excluded from this calculation (denoted as N/A) due to high sequence divergence (pS ≥ 0.75), which precludes reliable Ka/Ks estimation. The highest ratio was observed for *AcXYLP7/AcXYLP8* (0.51), while the lowest was found for *AcXYLP10/AcXYLP11* (0.18) (Table S3), implying the *XYLP* genes in kiwifruit evolved under a strong purifying selective pressure. The evolutionary relationships of orthologous *XYLP* genes were analyzed through comparative synteny among kiwifruit, *Arabidopsis*, and poplar genomes. The collinear analysis revealed that most *AcXYLP* genes had a high synteny with *XYLP* genes in other species (Figure 4B, Table S4). Compared to *Arabidopsis*, one additional *AcXYLP* orthologous gene was identified in poplar (Figure 4B), which may indicate a closer phylogenetic relationship with poplar.

### 3.4. GO Annotation and Protein–Protein Interaction Network Prediction of the AcXYLP Family Members

**Figure 5:**
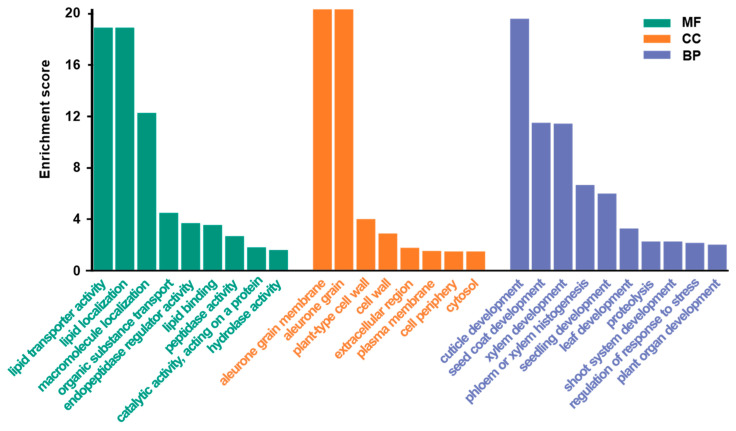
GO annotation of the AcXYLPs. Three functional categories were displayed: molecular functions (MF), cellular components (CC), and biological processes (BP).

To gain a preliminary understanding of the biological functions of the 28 AcXYLPs, gene ontology (GO) was employed for functional annotation. GO enrichment analysis revealed that these differentially expressed *AcXYLPs* were associated with multiple biological processes, cellular components, and molecular functions (Figure 5, Table S5). At the molecular-function level, the AcXYLPs were mainly involved in lipid transporter activity (GO:0005319), lipid localization (GO:0010876), lipid binding (GO:0008289) and catalytic activity on a protein (GO:0140096). At the biological-process level, the AcXYLPs were mainly involved in cuticle development (GO:0042335), xylem development (GO:0010089), seedling development (GO:0090351), leaf development (GO:0048366), shoot system development (GO:0048367) and regulation of response to stresses (GO:0080134). The most significant enrichment was observed at the cellular-component level, mainly enriched in the aleurone grain membrane (GO:0032578), plant-type cell wall (GO:0009505) and extracellular regions (GO:0005576) (Figure 5). These results indicated that the AcXYLPs were primarily involved in lipid transport, localization and binding, and may play significant roles in plant organ development and plant response to stresses.

**Figure 6:**
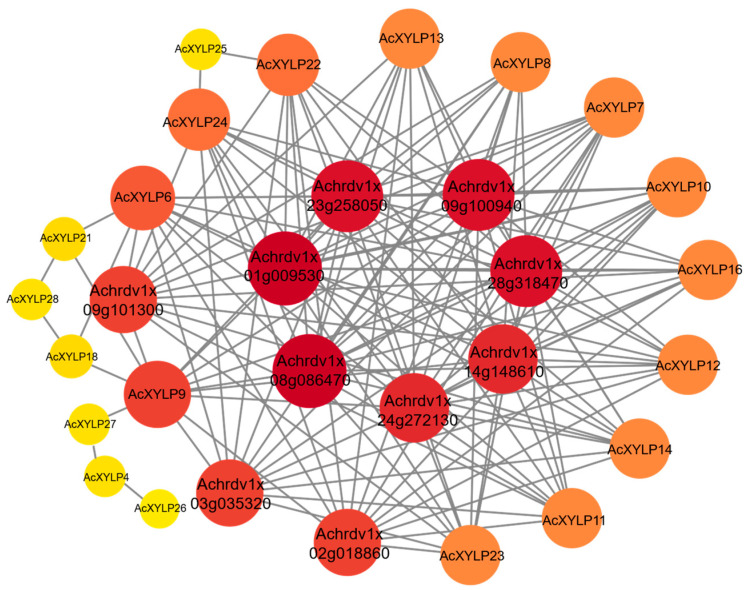
Protein–protein interaction network analysis of the AcXYLPs. The top ten highly enriched and targeted AcXYLP are shown; the darker and larger the color, the more highly enriched.

To better understand the potential biological functions of XYLPs in kiwifruit, the potential proteins interacting with AcXYLPs were analyzed using the STRING database V 12.0. The protein–protein interaction network was predicted and analyzed based on the related proteins in *A. chinensis*. According to the prediction, 20 of the 28 AcXYLPs established connections with its potential interacting proteins (Figure 6, Table S6). The results indicated that the *XYLP* family members, namely *AcXYLP6*, *AcXYLP7*, *AcXYLP8*, *AcXYLP9*, *AcXYLP10*, *AcXYLP11*, *AcXYLP12*, *AcXYLP13*, *AcXYLP14*, *AcXYLP16*, *AcXYLP22*, *AcXYLP23* and *AcXYLP24*, play crucial roles in plant growth and development (Figure 6). Specifically, these genes were predicted to be involved in the regulation of the transition of meristems from the vegetative to the reproductive phase, and also participated in the plant’s responses to auxin and ethylene (Figure 6, Table S6).

### 3.5. Cis-Acting Elements Present in the AcXYLP Gene Family Promoters

**Figure 7:**
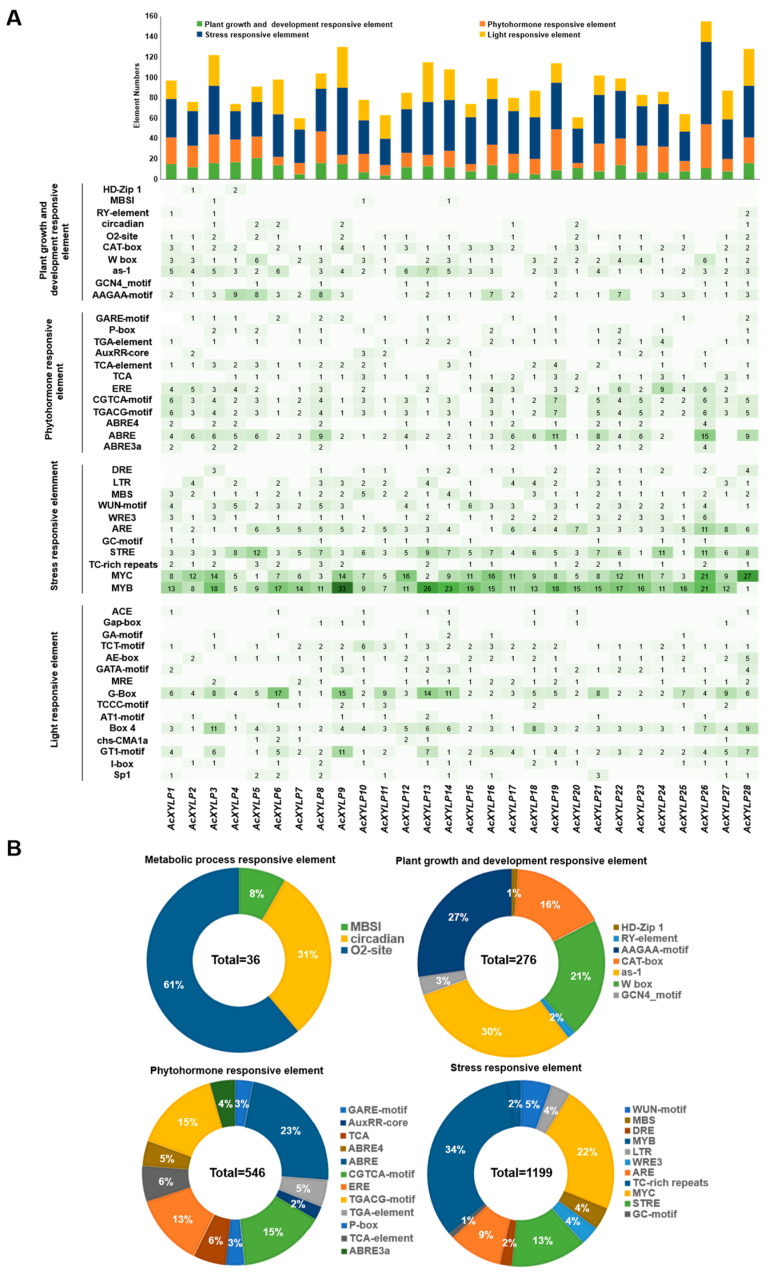
Distribution of *cis*-acting elements in the promoters of *AcXYLP* genes. (**A**) Heatmap showing the counts and distribution of *cis*-acting elements in the promoters of the *AcXYLP* genes. A darker color corresponded to a greater number of *cis*-acting elements. (**B**) Pie chart showing the proportion of different *cis*-acting elements based on their specific biological activities: metabolic-process-responsive elements, plant growth and development-responsive elements, phytohormone-responsive elements, and stress-responsive elements.

In order to investigate the putative roles of the *AcXYLP* genes in kiwifruit, the *cis*-acting elements in their promoter sequences were extracted and analyzed with plantCARE. Promoter analysis of the *AcXYLP* genes identified 2620 putative *cis*-acting elements, which were classified into 48 functional types (Table S7). According to the predicted biological functions, these *cis*-acting elements fall into four major groups: plant growth and development, phytohormone signaling, stress responses, and light-responsive elements. Each *AcXYLP* gene promoter contained various types of *cis*-acting elements, and the numbers differed (Figure 7). Among all the *cis*-acting elements, the stress-responsive elements accounted for the highest percentage at 45.8%, followed by light-responsive elements (21.5%), phytohormone-responsive elements (20.8%), and plant growth and development responsive element (11.9%). The methyl jasmonate (MeJA)-responsive elements and abscisic acid (ABA)-responsive elements constituted the largest proportions within the phytohormone-related category, accounting for 32% and 30%, respectively, with 175 and 164 identified in the 26 *AcXYLP* genes (Figure 7A). Furthermore, all of these genes contained salicylic acid (SA)-responsive elements (Figure 7A). The promoters of all *AcXYLP* genes contained MYB and MYC binding sites, which constituted 34% and 22% of the stress response elements, respectively (Figure 7A).

The plant growth and development category contained ten *cis*-acting elements. Six were implicated in key processes: differentiation of palisade mesophyll cells (HD-Zip 1), meristem expression (CAT-box), senescence expression (W box), shoot expression (as-1) and endosperm expression (GCN4_motif and AAGAA-motif) (Figure 7B), and the shoot expression (as-1) were extensively distributed in the promoters of 26 *AcXYLP* genes, with 82 occurrences (Figure 7A). Multiple *cis*-acting elements related to the phytohormone-responsive element category were found in *AcXYLP* gene promoters, including gibberellin (GARE-motif and P-box), auxin (TGA-element and AuxRR-core), salicylic acid (TCA-element and TCA), ethylene (ERE), MeJA (CGTCA-motif and TGACG-motif) and ABA (ABRE4, ABRE and ABRE3a) (Figure 7B). These phytohormone-responsive elements were widely present and can be found in almost every *AcXYLP* gene, especially the ABA-, MeJA-, and SA-responsive elements (Figure 7A). Among the stress-responsive elements, there are not only MYB- and MYC-responsive elements but also dehydration-responsive elements (DRE), low-temperature-responsive elements (LTR), drought-responsive elements (MBS), and wound-responsive elements (WUN-motif) (Figure 7B). The MYB- and MYC-responsive elements were closely associated with drought stress, salt stress, and cold stress [57,58,59]. The results above suggested that members of the *AcXYLP* gene family play important roles in plant responses to phytohormones and various stress conditions.

### 3.6. Expression Patterns of the AcXYLP Family Members in Different Tissues and Developmental Stages

**Figure 8:**
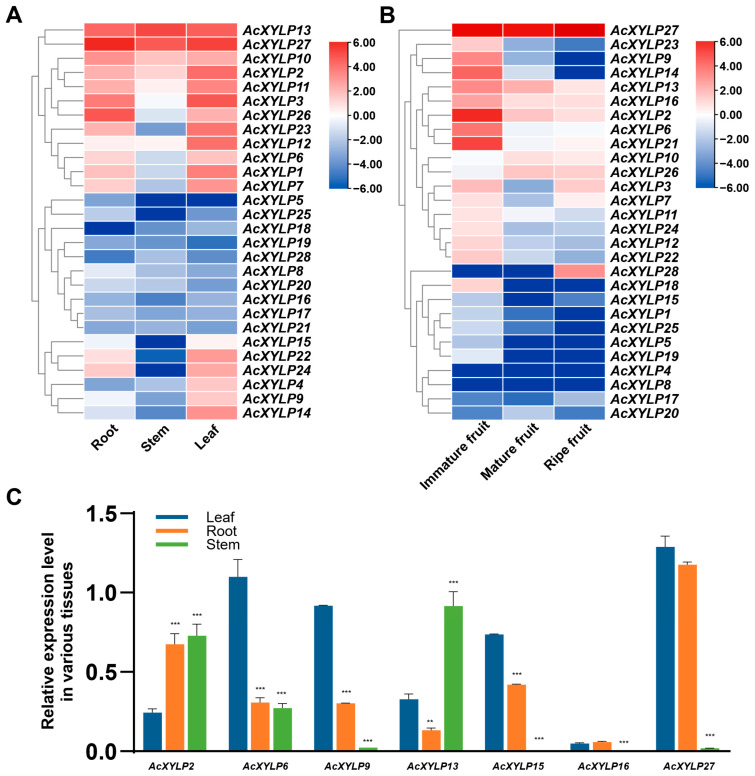
Tissue-specific expression profiles of the *AcXYLP* genes. RNA-seq transcriptomic data for tissue-specific expression patterns (**A**) PRJNA328414 and (**B**) PRJNA187369. (**C**) Quantitative real-time PCR amplification of seven *AcXYLP* genes in different organs. Results are presented as mean ± standard deviation (SD). *p* values of <0.01 (**) and <0.001 (***) were significant statistically.

Analysis of the gene expression patterns can provide important clues for elucidation of gene potential functions. Based on the RNA-seq datasets publicly available at NCBI Sequence Read Archive (SRA), the expression patterns of all 28 *AcXYLP* genes in different kiwifruit tissues were analyzed (Table S8). RNA-seq transcriptomic data for tissue-specific expression patterns was obtained under the accession of PRJNA328414 and PRJNA187369, encompassing vegetative organs such as roots, stems, and leaves, as well as three stages of fruit ripening: immature fruit, mature fruit, and ripe fruit (Figure 8A,B). A hierarchical cluster analysis was performed using the expression levels of *AcXYLP* genes. This analysis revealed that all 28 *AcXYLP* genes were expressed in at least one vegetative organ or reproductive developmental stage. Based on the transcriptome profiling data revealed by the PRJNA328414, *AcXYLP13* and *AcXYLP27* exhibited high expression levels across all vegetative organs (Figure 8A). *AcXYLP2*, *AcXYLP10* and *AcXYLP11* were abundantly expressed in roots and leaves but showed low expression levels in stems (Figure 8A). Nine genes (*AcXYLP1*, *AcXYLP3*, *AcXYLP6*, *AcXYLP7*, *AcXYLP12*, *AcXYLP22*, *AcXYLP23*, *AcXYLP24* and *AcXYLP26*) were primarily expressed in roots and leaves (Figure 8A). The expression levels of ten genes (*AcXYLP5*, *AcXYLP8*, *AcXYLP16*, *AcXYLP17*, *AcXYLP18*, *AcXYLP19*, *AcXYLP20*, *AcXYLP21*, *AcXYLP25*, and *AcXYLP28*) were relatively low in all vegetative organs, with some being undetected in specific tissues. Additionally, *AcXYLP4*, *AcXYLP9*, *AcXYLP14* and *AcXYLP15* were predominantly expressed in leaves (Figure 8A).

During the kiwifruit ripening process described in PRJNA187369, the genes *AcXYLP10*, *AcXYLP26* and *AcXYLP28* were found to be upregulated. The expression levels of 15 genes, including *AcXYLP2*, *AcXYLP3*, *AcXYLP6*, *AcXYLP7*, *AcXYLP9*, *AcXYLP11*, *AcXYLP12*, *AcXYLP13*, *AcXYLP14*, *AcXYLP16*, *AcXYLP18*, *AcXYLP21*, *AcXYLP22 AcXYLP23*, and *AcXYLP24*, were significantly reduced during fruit ripening (Figure 8B). It was worth noting that three genes (*AcXYLP2*, *AcXYLP13* and *AcXYLP27*) were expressed at high levels in both vegetative and reproductive tissues (Figure 8A,B). This showed that these *AcXYLP* genes might play crucial roles in all tissues throughout the developmental stages of kiwifruit. To confirm the expression profiles of *AcXYLP* genes in different tissues, primers were designed for qRT-PCR amplification of the 28 *AcXYLP* genes. Due to non-specific amplification, with the trial of three primer pairs for each gene, the expression patterns of only seven genes were successfully obtained: *AcXYLP2*, *AcXYLP6*, *AcXYLP9*, *AcXYLP13*, *AcXYLP15*, *AcXYLP16*, and *AcXYLP27* (Figure 8C). Fortunately, these seven genes were distributed across five clades, and these genes were further used in subsequent experiments. Gene-specific primers used in qRT-PCR are listed in Table S1 and melting curves are shown in Figure S4. Samples were collected from 3-month-old ‘Hong Yang’ soil-grown plants, and the expression levels of these seven genes during the vegetative growth stage were detected, which were highly consistent with results previously described in RNA-seq (Figure 8A,C).

### 3.7. Expression Profiles of the AcXYLP Family Members in Response to Abiotic Stresses and Phytohormone Treatments

**Figure 9:**
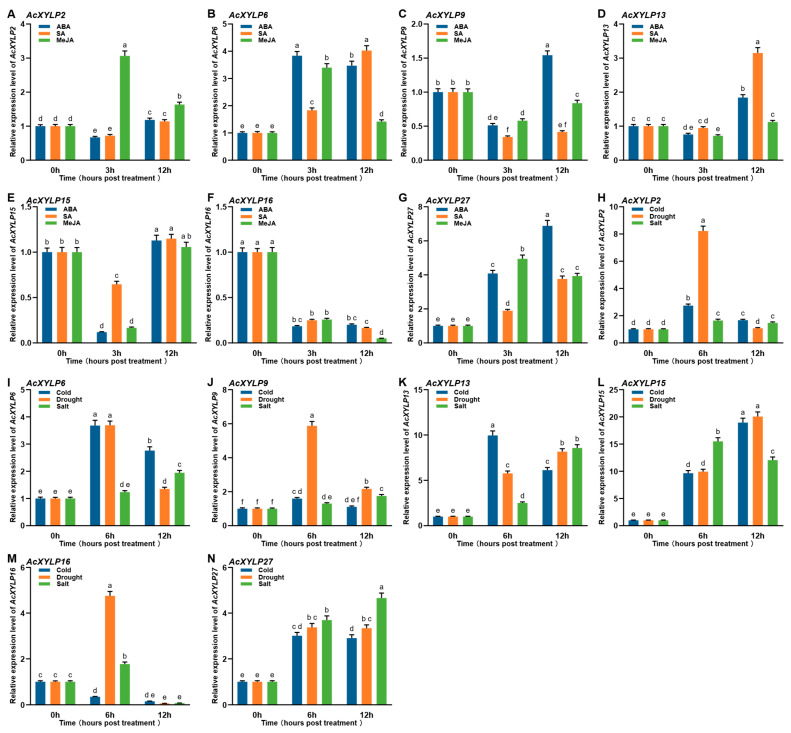
Expression profiles of selected *AcXYLP* genes following phytohormone application and abiotic stress exposure. The relative expression levels of *AcXYLP2*, *AcXYLP6*, *AcXYLP9*, *AcXYLP13*, *AcXYLP15*, *AcXYLP16*, and *AcXYLP27* are displayed for phytohormone treatments (**A**–**G**) and abiotic stresses (**H**–**N**). Values are expressed as mean ± standard deviation (SD). A two-way ANOVA was performed, with significant effects further analyzed by Tukey’s Honest Significant Difference (HSD) post hoc test for pairwise comparisons. Different superscript letters indicate statistically significant differences at *p* < 0.05.

Previous studies have shown that *XYLP* genes play roles in plant responses to multiple phytohormone treatments and abiotic stresses [30,31]. To determine whether *AcXYLP* genes also function in abiotic stresses and phytohormones, the expression levels of seven genes (*AcXYLP2*, *AcXYLP6*, *AcXYLP9*, *AcXYLP13*, *AcXYLP15*, *AcXYLP16*, and *AcXYLP27*) were examined in kiwifruit plants subjected to cold, drought, salt, ABA, SA, and MeJA treatments (Figure 9). Expression pattern analysis revealed that most *AcXYLP* genes were significantly upregulated in response to cold, drought, and salt stress, compared to untreated controls, except *AcXYLP2* and *AcXYLP16* (Figure 9A–G). Under salt stress, the expression of *AcXYLP2* showed an initial downregulation followed by upregulation (Figure 9A). The expression of *AcXYLP16* was downregulated under cold stress, and exhibited an initial upregulation followed by downregulation under drought and salt stress (Figure 9B). Notably, the expression levels of *AcXYLP13*, *AcXYLP15* and *AcXYLP27* genes were significantly upregulated under three stress conditions: cold, drought, and salt stress (Figure 9D,E,G).

Most *AcXYLP* genes showed similar expression profiles after exposure to phytohormone treatments (Figure 9H–N). The expression of *AcXYLP6* and *AcXYLP27* was upregulated under ABA, SA and MeJA treatments, and the expression of *AcXYLP16* was downregulated (Figure 9I,M,N). The expression of *AcXYLP13* and *AcXYLP15* was first downregulated and then upregulated by ABA, SA and MeJA treatments (Figure 9K,L). *AcXYLP2* was initially downregulated by ABA and SA treatments, then upregulated, while it was upregulated by MeJA treatment (Figure 9H). *AcXYLP9* was initially downregulated by ABA and MeJA treatments, then upregulated, while it was downregulated by SA treatment (Figure 9J). All these results indicated that all seven *AcXYLP* genes participated in plant response to abiotic stresses and phytohormone treatments, with *AcXYLP13*, *AcXYLP15* and *AcXYLP27* potentially playing significant roles in enhancing plant tolerance to abiotic stresses.

## 4. Discussion

### 4.1. Molecular Characteristics and Evolutionary Analysis of the AcXYLP Genes

In this study, 28 *AcXYLP* genes were identified in the genome of *Actinidia chinensis*. These AcXYLPs contained at least one PAST-rich (PAST residues ≥ 35%) region and a highly conserved nsLTP domain (C_1_-X-C_2_-X-P-X-C_3_C_4_-X-C_5_XC_6_-X-C_7_-X-C_8_) (Figure 3B), which was in agreement with the characteristics of XYLPs in *Arabidopsis* [23] and poplar [30] plants. Notably, there were significant differences in the physicochemical properties of AcXYLPs, including variations in the number of amino acids, isoelectric points, and instability indices (Figure 3A). Despite these differences, all XYLPs were in silico-predicted to be hydrophobic, consistent with their roles in transmembrane signaling transduction (Table 1). In addition, the differences in the physicochemical parameters of AcXYLP might be attributed to the structural diversity of the *XYLP* genes. In agreement with previous reports, the members of *AcXYLP* gene family contained one to four exons and zero to three introns, and genes within the same phylogenetic clade exhibited similar gene structures [23,29,30,31] (Figure S1). *AcXYLP4*, *AcXYLP25*, and *PeXYLP1* exhibited a reduced number of exons; each possessed only a single exon [31] (Figure S1). This reduction might be due to the disruption of splicing sites caused by the insertion of transposable elements. Additionally, the subcellular localization of the AcXYLPs was in silico-predicted to be on the plasma membrane, and this was proved on AcXYLP1 and AcXYLP27 in *Arabidopsis* (Figure 2), suggesting a potential role in transmitting intercellular signals and inducing differentiation into vascular tissues.

To elucidate the evolutionary relationships of the *XYLP* gene family, the phylogeny of kiwifruit was compared with that of *Arabidopsis* and poplar. It was found that, as a woody plant, the genes of the *XYLP* family in kiwifruit had likely evolved independently, paralleling the findings in poplar [30]. There are 28 *AcXYLP* genes in the kiwifruit genome, a number higher than the *XYLP* genes in *Arabidopsis* (13) [23], rice (21) [29], and moso bamboo (23) [31], but lower than the 31 *XYLP* genes present in poplar [30]. Gene duplication plays a fundamental role in the expansion and development of plant gene families during evolution, contributing to the generation of novel genes and the diversification of gene functions [56]. The kiwifruit genome contained 29 chromosomes, with 28 *AcXYLP* genes randomly distributed across 18 of these chromosomes (Figure 4A). Notably, the distribution of *AcXYLP* genes did not correlate with chromosome size, and 11 chromosomes did not harbor any *AcXYLP* genes (Figure 4A). These findings suggested that the *AcXYLP* gene family in kiwifruit may have undergone segmental loss events during evolution. In *A. chinensis*, all identified duplicated *XYLP* gene pairs originated from segmental duplication events (Table S4). The absence of tandemly duplicated gene pairs suggested that segmental duplication was the dominant force shaping the kiwifruit *XYLP* family. While *XYLP* expansion in other species commonly involves both segmental and tandem events [29,30,31], kiwifruit appeared to have followed a divergent evolutionary path, likely shaped by its specific long-term adaptive pressures.

### 4.2. Prediction of the Potential Biological Functions of the AcXYLP Genes

*Cis*-acting elements are reported to be pivotal regulators of transcriptional patterns and levels [60]. Analysis of the *AcXYLP* gene promoters revealed an enrichment of *cis*-acting elements responsive to plant growth and development, stress, phytohormones, and light, underscoring their potential roles in these key biological processes. For instance, there are abundant elements related to meristem (CAT-box), shoot (as-1), and endosperm (GCN4_motif and AAGAA-motif) development (Figure 7B). Additionally, the *AcXYLP* genes contained many phytohormone-responsive elements, including those for auxin, ethylene, and gibberellin (Figure 7A), which played crucial roles in various physiological processes, such as seed germination [61,62], root development [63,64], stem elongation [65,66], vascular differentiation [67], and fruit ripening [68].

In addition to elements related to stress responses, such as dehydration, salt stress response (DRE), low-temperature response (LTR), wound response (WUN-motif), and drought response (MBS), the *AcXYLP* genes also contained a significant number of MYB and MYC elements closely associated with low-temperature, drought, and salt stress [57,58,59] (Figure 7B). It was suggested that *XYLPs* in kiwifruit played key roles in regulating plant growth, development, and responses to abiotic stresses. GO enrichment analysis identified a substantial number of *AcXYLP* genes associated with lipid transport and catalytic activity. Furthermore, these *AcXYLP* genes were also involved in the development of leaves, xylem, and shoots, as well as in the plant’s response to environmental stresses (Figure 5). The analysis of the protein–protein interaction network revealed that many AcXYLPs participated in plant growth and hormone signaling (Figure 6), which aligned with the findings from the GO enrichment analysis (Figure 5).

### 4.3. Expression Profile Reveals the Potential Roles of the AcXYLP Genes in Abiotic Stresses and Phytohormone Responses

According to relevant studies, multiple XYLPs have been found to be involved in the development of plant vascular systems [22,26,27,29,30]. In *Arabidopsis*, the *XYLP* double-knockout mutant (*xyp1 xyp2*) displayed discontinuous veins and vessels in both leaves and roots, which suggested that the absence of XYLPs significantly impaired the formation of vascular tissues [22]. The defects observed in the internodes of the rice *xylp7* mutant and the leaf veins of *35Spro::PtXYLP1* overexpressing plants indicated that XYLPs play a crucial role in the development of the vascular system [29,30]. In this study, two genes, *AcXYLP13* and *AcXYLP27*, exhibited high expression levels in all vegetative tissues (Figure 8A,C). The remaining 16 genes were abundantly expressed in at least one vegetative tissue, with 12 genes being mainly expressed in roots and leaves, while 4 genes were mainly expressed in leaves (Figure 8A). These results indicated that AcXYLPs played a significant role in plant vegetative growth, which was similar to findings described in poplar and moso bamboo [30,31]. As kiwifruit matures, the expression of most *AcXYLP* genes was downregulated. However, the expression levels of *AcXYLP10*, *AcXYLP26*, and *AcXYLP28* were significantly upregulated, while *AcXYLP27* consistently maintained a high expression level (Figure 8B). These findings suggested that members of the *AcXYLP* gene family play a crucial role in regulating the fruit ripening process.

Abiotic stress triggers the expression of *XYLP* genes. For example, *OsXYLP7* is induced under drought, salt, and cold stress [29], and *PeXYLP2* and *PeXYLP9* are activated by PEG and NaCl [31]. The *AcXYLP* genes exhibited diverse expression patterns in response to abiotic stress and various phytohormone treatments. After exposure to cold, drought, and salt stress, the expression of the seven *AcXYLP* genes, except for *AcXYLP16*, was found to be upregulated. Notably, *AcXYLP13*, *AcXYLP15*, and *AcXYLP27* displayed significant increases in their expression levels (Figure 9A–G). Phytohormone treatments also induce the expression of *XYLP* genes, with *PtXYLP1* and *PtXYLP2* being induced by NAA, 6-BA, and GA treatments [30]. The majority of *AcXYLP* genes showed modulated expression at different time points during phytohormone treatments. It was noteworthy that under ABA stress, the expression levels of genes *AcXYLP2*, *AcXYLP6*, *AcXYLP9*, *AcXYLP13*, *AcXYLP15*, and *AcXYLP27* were significantly upregulated (Figure 9H–N). This upregulation was also observed in response to cold, drought, and NaCl abiotic stresses (Figure 9A–G). These findings might be attributed to the role of ABA, as a stress hormone, which played a crucial role in mediating various abiotic stress responses in plants [69].

Consistent with *cis*-acting element predictions, the promoters of *AcXYLP* genes contained multiple MYB and MYC elements. The results implicated the kiwifruit *XYLP* gene family in crucial abiotic stress response pathways.

## 5. Conclusions

This study identified and characterized members of the *XYLP* gene family in the kiwifruit genome using bioinformatics methods to elucidate the structural and functional roles. A total of 28 *AcXYLP* gene family members were identified, which were unevenly distributed across 18 chromosomes. The expansion of the *AcXYLP* gene family was suggested to have primarily originated from segmental duplication events and had undergone strong purifying selection pressure throughout evolution. Based on the phylogenetic analysis, the *AcXYLP* genes were grouped into five clades, exhibiting similar gene structures and conserved motif compositions within each clade. Subsequently, the potential biological functions of *AcXYLP* genes were investigated in silico through analysis of promoter *cis*-acting elements, gene ontology annotations, and protein–protein interaction networks. This analysis indicated that *AcXYLP* genes might play pivotal roles in the regulation of growth, development, hormone regulation, and abiotic stress responses of *A. chinensis*. RNA-seq transcriptomic analysis, complemented by qRT-PCR validation, suggested the involvement of *XYLP* genes in the growth of vegetative tissues and the fruit ripening process in kiwifruit. Moreover, the *AcXYLP* genes were implicated in the plant’s response to phytohormones (e.g., ABA, SA, and MeJA) and abiotic stresses (e.g., cold, drought, and salt). Overall, this study provided valuable insights into the role of the *XYLP* gene in the growth and development of kiwifruit, as well as its response to abiotic stress. This research established a robust foundation for future functional validation and applied studies in molecular breeding.

## Figures and Tables

**Table 1:** Physicochemical properties of *AcXYLP*.

Gene Name	Gene ID	Amino Acids	MW (kDa)	pI	Aliphatic Index	Instability Index	Hydropathicity	Subcellular Localization	Pred. GPI-Anchored	Pred. GPI-Anchored Likelihood
*AcXYLP1*	Achrdv1x19g206970	179	17.47	7.50	93.30	45.22	0.677	Cell membrane	Yes	0.638
*AcXYLP2*	Achrdv1x07g069520	177	17.44	6.03	99.27	56.50	0.601	Cell membrane	Yes	0.371
*AcXYLP3*	Achrdv1x06g063540	178	17.32	6.50	91.12	50.40	0.626	Cell membrane	Yes	0.689
*AcXYLP4*	Achrdv1x11g116950	153	16.22	6.02	93.07	41.20	0.653	Cell membrane	no	0.893
*AcXYLP5*	Achrdv1x21g226030	196	19.40	3.79	82.81	57.65	0.610	Cell membrane	Yes	0.508
*AcXYLP6*	Achrdv1x28g317560	200	19.33	4.18	95.15	57.60	0.465	Cell membrane	Yes	0.312
*AcXYLP7*	Achrdv1x20g214560	182	18.77	8.42	81.54	51.36	0.203	Cell membrane	Yes	0.203
*AcXYLP8*	Achrdv1x29g328370	193	19.91	9.11	80.88	51.51	0.111	Cell membrane	Yes	0.396
*AcXYLP9*	Achrdv1x23g257850	210	21.87	5.62	98.52	58.16	0.380	Cell membrane	Yes	0.400
*AcXYLP10*	Achrdv1x24g268350	171	17.22	7.48	79.30	59.38	0.224	Cell membrane	Yes	0.340
*AcXYLP11*	Achrdv1x03g038080	171	17.10	5.04	77.60	59.03	0.177	Cell membrane	Yes	0.297
*AcXYLP12*	Achrdv1x08g081880	192	20.36	7.47	95.52	47.82	0.131	Cell membrane	Yes	0.276
*AcXYLP13*	Achrdv1x15g166120	196	21.10	8.35	88.11	60.28	0.037	Cell membrane	Yes	0.396
*AcXYLP14*	Achrdv1x26g299150	172	17.96	8.54	80.00	44.22	−0.033	Cell membrane	Yes	0.437
*AcXYLP15*	Achrdv1x24g268360	179	17.98	7.51	79.61	76.75	0.331	Cell membrane	Yes	0.552
*AcXYLP16*	Achrdv1x28g319270	187	19.64	5.67	93.85	36.23	0.117	Cell membrane	Yes	0.509
*AcXYLP17*	Achrdv1x20g218800	195	19.89	7.46	81.13	44.07	0.040	Cell membrane	Yes	0.607
*AcXYLP18*	Achrdv1x07g077000	194	20.62	5.06	79.95	36.03	0.134	Cell membrane	Yes	0.397
*AcXYLP19*	Achrdv1x26g288760	182	18.65	7.58	97.64	62.15	0.355	Cell membrane	Yes	0.496
*AcXYLP20*	Achrdv1x28g318050	195	20.13	8.93	97.54	64.15	0.303	Cell membrane	Yes	0.495
*AcXYLP21*	Achrdv1x13g145110	188	19.28	6.69	80.53	45.24	0.169	Cell membrane	no	0.385
*AcXYLP22*	Achrdv1x17g183400	208	21.09	8.15	91.54	60.73	0.527	Cell membrane	Yes	0.663
*AcXYLP23*	Achrdv1x17g183410	197	19.32	5.05	87.72	50.93	0.444	Cell membrane	Yes	0.369
*AcXYLP24*	Achrdv1x24g268370	202	20.50	7.51	89.01	54.57	0.417	Cell membrane	Yes	0.804
*AcXYLP25*	Achrdv1x11g123850	159	16.92	6.78	93.84	40.77	0.236	Cell membrane	Yes	0.469
*AcXYLP26*	Achrdv1x15g163330	143	14.90	4.77	83.29	40.95	0.424	Cell membrane	Yes	0.663
*AcXYLP27*	Achrdv1x14g154760	149	15.35	7.45	94.30	41.05	0.330	Cell membrane	Yes	0.638
*AcXYLP28*	Achrdv1x16g174090	193	20.08	6.52	78.39	56.22	0.125	Cell membrane	Yes	0.281

## Data Availability

All data generated or analyzed during this study are included in this published article and its Supplementary Files.

## Supplementary Materials

The following supporting information can be downloaded at: https://www.mdpi.com/article/10.3390/biology15030264/s1, Supplementary Figure S1: Gene structure and clade information of *XYLP* genes from kiwifruit, *Arabidopsis* and poplar. Genes were clustered and shown due to different clades. Clade information was shown in different colors on the left side. Upstream/downstream regions, CDS, and introns were displayed by colored boxes and black lines. The abbreviations “*Ac*” for *Actinidia chinensis*, “*At*” for *Arabidopsis thaliana*, “*Pt*” for *Populus trichocarpa* were placed before the gene family name (*XYLP*). Supplementary Figure S2: 3D structure prediction of AcXYLP proteins, applied with SWISS-MODEL Workspace (https://swissmodel.expasy.org/). Supplementary Figure S3: 3D structure prediction of conserved nsLTP domains, applied with SWISS-MODEL Workspace (https://swissmodel.expasy.org/). Supplementary Figure S4: Melting curves for qRT-PCR amplification. Rows show the results for: (A) *AcActin*, (B) *AcXYLP2*, (C) *AcXYLP6*, (D) *AcXYLP9*, (E) *AcXYLP13*, (F) *AcXYLP15*, (G) *AcXYLP16*, and (H) *AcXYLP27*. Table S1: Gene-specific primers used in quantitative real-time PCR; Table S2: Protein backbones of XYLPs in kiwifruit; Table S3: Ka-Ks-selected result; Table S4: Gene collinearity pairs; Table S5: GO annotation; Table S6: String analysis; Table S7: *cis*-acting elements in the promoter of *AcXYLPs*; Table S8: Gene expression profiles of *AcXYLPs*; Table S9: Detailed information of 3D structure prediction of AcXYLP proteins; Table S10: Detailed information of 3D structure prediction of conserved nsLTP domains.

## Abbreviations

The following abbreviations are used in this manuscript:

ABA	abscisic acid
AG	arabinogalactan
AGP	Arabinogalactan protein
ANOVA	analysis of variance
BP	biological processes
CC	cellular components
DAP	days after pollination
FLA	fasciclin-like AGP
HMM	hidden Markov model
HSD	Honestly Significant Difference
GFP	green fluorescent protein
GO	gene ontology
GPI	glycosylphosphatidylinositol
MeJA	methyl jasmonate
MF	molecular functions
MS	Murashige and Skoog
MW	molecular weight
nsLTP	non-specific lipid-transfer protein
pI	isoelectric point
PLA	plastocyanin-like AGP
PEG	polyethylene glycol
RFP	red fluorescent protein
SA	salicylic acid
SCAMP	secretory carrier membrane protein
SRA	sequence read archive
TPK	two pore K^+^
TPM	transcripts per million
XYLP	xylogen-like protein
