# Supplementary material for: Identification, Characterization and Expression Profiles of Xylogen-like Gene Family in Kiwifruit in Different Developmental Tissues and Under Various Abiotic Stresses
Source: Biology (Basel). 2026 Jan 31;15(3):264. doi: 10.3390/biology15030264 (PMC12896894; doi:10.3390/biology15030264)
Supplement: Supplementary file 1 [file biology-15-00264-s001.zip › Supplementary Figure Caption.pdf]

Supplementary Figure S1. Gene structure and clade information of *XYLP* genes from kiwifruit, *Arabidopsis* and poplar. Genes were clustered and shown due to different clades. Clade information was shown in different colors on the left side. Upstream/downstream regions, CDS, and introns were displayed by colored boxes and black lines. The abbreviations "*Ac*" for *Actinidia chinensis*, "*At*" for *Arabidopsis thaliana*, "*Pt*" for *Populus trichocarpa* were placed before the gene family name (*XYLP*).

Supplementary Figure S2. 3D structure prediction of AcXYLP proteins, applied with SWISS-MODEL Workspace (https://swissmodel.expasy.org/).

Supplementary Figure S3. 3D structure prediction of conserved nsLTP domains, applied with SWISS-MODEL Workspace (https://swissmodel.expasy.org/).

Supplementary Figure S4. Melting curves for qRT-PCR amplification. Rows show the results for: (A) *AcActin*, (B) *AcXYLP2*, (C) *AcXYLP6*, (D) *AcXYLP9*, (E) *AcXYLP13*, (F) *AcXYLP15*, (G) *AcXYLP16*, and (H) *AcXYLP27*.
