# Supplementary material for: Identification, Characterization and Expression Profiles of Xylogen-like Gene Family in Kiwifruit in Different Developmental Tissues and Under Various Abiotic Stresses
Source: Biology (Basel). 2026 Jan 31;15(3):264. doi: 10.3390/biology15030264 (PMC12896894; doi:10.3390/biology15030264)
Supplement: Supplementary file 1 [file biology-15-00264-s001.zip › Table S1. Gene-specific primers used in quantitative real-time PCR.pdf]

Table S1. Gene-specific primers used in quantitative real-time PCR

Primer Name	Sequence (5' to 3')
AcACTIN_F	TGCATGAGCGATCAAGTTTCAAG
AcACTIN_R	TGTCCCATGTCTGGTTGATGACT
AcXYLP2_F	CTACTTCGCCAGCGGGTG
AcXYLP2_R	ACCTGAGCTTCTCGGAGC
AcXYLP6_F	CCCCTCCCGTTAGCTTGTG
AcXYLP6_R	CTGAGGCTCCATTGGGGC
AcXYLP9_F	CGTGTCGGACTGCCTGAG
AcXYLP9_R	GCAGAGGCAGATGGGGTG
AcXYLP13_F	GGCTTGCCCTCTGTTTGC
AcXYLP13_R	TTGGGCTGCTGGATGAGG
AcXYLP15_F	GGCTCAGTCAGGGTGCAC
AcXYLP15_R	CAACACGAAGAAGGCGGG
AcXYLP16_F	CTGAGTGCCCTGCTCTCC
AcXYLP16_R	ATTCGTGGCTGGGGTAGTG
AcXYLP27_F	AGGGGCAATCTTCCACGC
AcXYLP27_R	TCGTCACGGCCTCTCTCA
