# Supplementary material for: Identification, Characterization and Expression Profiles of Xylogen-like Gene Family in Kiwifruit in Different Developmental Tissues and Under Various Abiotic Stresses
Source: Biology (Basel). 2026 Jan 31;15(3):264. doi: 10.3390/biology15030264 (PMC12896894; doi:10.3390/biology15030264)
Supplement: Supplementary file 1 [file biology-15-00264-s001.zip › Table S2. Protein backbones of XYLPs in kiwifruit.pdf]

Table S2. Protein backbones of XYLPs in kiwifruit

Name	Locus	Protein backbones
AcXYLP1	Achrdv1x19g206970	MAMNTMTLLLSVLAISVVANAGSPAPAVD**CSTVVVSMADCLTYVTGASSVKKPEGNCCSGLKTVLKSNPQCLCETFKNSAQFGITLNVTKALSLPAACHVSAPSVSNC**GLSLVAGAAPVTSPVSAVSPIPGGPTSPAGSPAGGNDLAPAPAPMMASSATPAFLVGSFLAALLAASFSFI
AcXYLP2	Achrdv1x07g069520	MAIHAVTLSLLIPILSLCFSGTVQSTTAPAPAVD**CSGVFLSLADCLSYVTGNDSVKKPEGTCCSGLKTVLKTEPECLCEAFKSSAQLGVTLNITRALALPSACHLSAPSVSNC**GLSIGGAPATSPAGALSPSTPTSVPGVSEITPASAPRSSGSVSPATSIVSLALALVFASFSSI
AcXYLP3	Achrdv1x06g063540	MAMNTMNLILLSVLAISFVANAGSPAPAAD**CSTVVLSMADCLTYVTGGSGVTKPEGNCCSGLKTVLKSNPQCLCETFKNSAQFGITLNVTKALSLPAACHVSSPSVSNC**GLSLVAGAAPATSPVSAVSPSPIAGGPTSPAGGNELAPAPAPAVTSSATPAFSVGSFLAALLAASFSFL
AcXYLP4	Achrdv1x11g116950	MAIHAVTLSLLIPILSLCFSGTVQSTTAPAPAVD**CSGVFLSLADCLSYVTGNDSVKKPEGTCCSGLKTVLKTEPECLCEAFKSSAQLGVTLNITRALALPSACHLSAPSVSNC**GCESFNFFFLAFFLGFLHNFPFVLCGSRFEHFCFVSNLST
AcXYLP5	Achrdv1x21g226030	MATMSYVSLAFCILATWALVSVSGAQSLAAAPSPAGAD**CTSLIYNMVDCISYLSVGSNQMTPDPACCSGFKTVLETNAACICEGFKSSVDLGIAIDMSKAMALPSACGVSAPSISSC**DVSSAPGAAPAFPPAANDAPAPESAEPPAVNASAPTTTGGGIVNEVPAPSPLSSGAYPVVFSSAVYVSIMFASFFSVLA
AcXYLP6	Achrdv1x28g317560	MTPATPIAGKMIVTATALLAVAVLVSGAAAPAPAPESGSGSGPVVQSPEPVSGPAPGPGGID**CISSLANVSDCLTYVEAGSNLTKPDEGCCPALAGLVESEPICLCELVAGNATESLGIEINMKKALKLPSVCGVQTPPVSLC**SLAGYPVPVPMPSPGPGGMAPESGSNNGSSPNGASGIAASVQLPILVASLTTLLFFG
AcXYLP7	Achrdv1x20g214560	MAHQRVRTIIVSQAKTFAYLHTILAMVIVLWTGATAQSSD**CTSALISMSPCLNYITGNSSTPSSGCCQQLASVVRSQPQCLCEAVNGGGSSMGIKVNQTQALALPGACNVQTPSISRC**NAASPVDSPARTTQAPNTIPSGTGSKTVPSTEDGSSDASSARMTVPLLFFLLFIASYASTSTIC
AcXYLP8	Achrdv1x29g328370	MAHHQRTGMGLASRAKTFAYLHTILATVIMLWTVVTAQSSN**CTKALISMSPCLNYITGNPSTPTSRCCQQLASVVRSQSQCLCEVLNGGGSSMGINVNQTQAVALPGACNVQTPPISRC**NVASPADSPAGTPQSPNTITSGTGSKTVPSTKDGSKTVPLTKDGLSDASSTRMTVSLLFLLLFIASSASTSTIC
AcXYLP9	Achrdv1x23g257850	MPLALLFTVPHYSPILSPKQLRSDRSRARERTMASIRFTAILGTVMAVAVLLVGASTVQPPPVAAPGPSPLD**CKSYLLNVSDCLSYVEADSNATEPERGCCPELAGLVETHPICLCQLLTNTPRFGIQISLSKALNLPSACGLITTPSTNMC**SGAGYAIGVSMPSEAPGLAGSPASENNDSGASSIAVSQIISLFLVCLPIPFLSTFFST
AcXYLP10	Achrdv1x24g268350	MASRAIGTGLVVVVVAVLWAGAAAQSSD**CTSVLISMSPCLNYISGNSSTPSSGCCSQLASVVRSKPQCLCEVLNGGGSSLGIDVNQTRALALPRACNVQTPPLSRC**KAASPADSPSVTPESPNTESSGTGSKTVPSTDSGSTPSDAGSTKMTFSLLFFLLFIASYPSTSAF
AcXYLP11	Achrdv1x03g038080	MASRVIGTSLVVVVVAALWAGAAAQSSD**CTSALISMSPCLNYISGNSSTPSSGCCSQLSNVVRSKPQCLCEVLNGGGSSIGIDVNQTQALALPGACNVQTPPLSRC**NAASPANSPSGTPESPNTEPSGNGSKTVPSTDSGSTPSDASSTKMTVSLLFFLLFIASYASTTTF
AcXYLP12	Achrdv1x08g081880	MDDHFTRVQLPLIFLTIYVILSCASSDPAKDREE**CTEQLVGLATCLPYVGGNAKAPTPDCCSGLKQVLKTNKKCLCVIIRDRNDPELGLTINVTLALGLPSVCNAPANVSQC**PALLKMAPNSPDAQIFYQLGHASASGPTSSPVANVKTNTTSGSGTSGQEKSGGNFSAQRWLGWQVIACALYIWVSIISLI
AcXYLP13	Achrdv1x15g166120	MDDHFTRVQLPLIFLTISMILSCASSDPAKDRKE**CTEQLVGLATCLPYVGGNAKAPTPDCCSGLKQVLKTNKKCLCVIIRDRNDPELGLTINVTLALGLPSVCNAPANVSQC**PALLQMPPNSPDA

		QIFYQFGNASSSSPTSSPSATGNANATVGPEKSGGRDLSGH<br>RWLRWEVIACGLSIWLFISRFLFIKFGWPP
AcXYLP14	Achrdv1x26g299150	MVGLASSDLAKDRQECADQLVGLATCLPYVGGQAKAPTLD<br>CCSGLKQVLKASKKCLCILVKDRNDPSLGLKINATLALSLPT<br>TCHAPSNVSECPALLHLAPNSTDAKVFEDFANSSKRSNSTTP<br>ATNGNSTSTGSTNTAKVKSDGGRGQRWLVFEMVCVAIFSM<br>VSSSCTSF
AcXYLP15	Achrdv1x24g268360	MPSNWVVALVLMVWAGGARAQSGCTNVLIGMAPCLNYVTG<br>RSPSPPSSCCSQLASVVQSQPQCLCAALSGGGSAFGININQ<br>TLALALPGACKVQTPPVSKCDAVNNGPATAPVSSPEGMPA<br>ETSTEAPGSPVSSPISGGSKAVPSNGATSHGRSIEMMPFQL<br>VTLFLFMASYASISISF
AcXYLP16	Achrdv1x28g319270	MDSKCVTCILVLTMVGLASSDLAKDRQECADQLVGLATCLP<br>YVSGEAKAPPVDCCSGLKQVLKDSKKCLCILVKDRNDPSL<br>GLKINATLALSLPTICNAPANLSECPALLHLAPNSTDAKVFED<br>FENSSKGSNSTTPATNVEGNSTSNGSTSTAEVKSDGGRGK<br>RWLVVEMVCVAIFSMVIHTLLSM.
AcXYLP17	Achrdv1x20g218800	MRVSKSLKMLPILAMLVAAVAVAVAVATMEEDEKDCADQLA<br>NLAACIPYVSGTAKKPTPQCCQDTQKVKSAKPKCLCVLIKE<br>STDPSMGLPVNTTLALQMPSQCNIDAKISDCPTLLNLSPDSP<br>DAKIFKSGSSTSSTTDAQPTPVSASSGTSSSSSGTGSGSDS<br>KATPTSNGVAKMSGSSLAMTSLTSLAWLLI.
AcXYLP18	Achrdv1x07g077000	MRTQSIVHIMIMGLFLGLYSVGAATVGEQCSNDFEKVATCLN<br>YATGKAEAPTKECCASVTEIKDSDPVCLCYIIQQTHDGGEQ<br>VKSLGIQEARLLQLPTACKLTNASASNCPKLLNLTVSSPDYE<br>FFTNISSGSSTTPSTTPSTTTGTSLPTTTTDGSNGPRHGPQL<br>AGLVSIAITTFFFFFFCAYPLEVVSIF.
AcXYLP19	Achrdv1x26g288760	MASPHPLSITATTFLTLLSLTLSQTPGGMSPAIAECGTSLLPL<br>APCAPFVQGRAAVPPQPCCGGLHQLYHQQPNCICLLLNDS<br>TLSSFPINTTLALQLPLLCNLQLDRSTCSGVPLPPSSPASQV<br>SFGTTTNSNSAASPTVTLGPRSTIIGFGQSGGARLNMESHLV<br>VTAAAAILCTKLLSH.
AcXYLP20	Achrdv1x28g318050	MSSLHSLPPKALTSLLLLFLTALIPHITLSQNPRGTSPPGPTIS<br>NCGPRLLPLVPCTPFVQGIAPTPARPCCDGLKQVNNQEPSC<br>LCLLLNGTALSSFPINPTLAPQLPRLCNLEVDISACSGVPSP<br>SSAPVSQVSSRTHPNSTIAASPTVTVAPRTSIMGLGFGQSSG<br>TKLKMNGVMLVAAMAFMSLEVLQSPA.
AcXYLP21	Achrdv1x13g145110	MRSQTLASFAAAAAVFLCTAAVGGGAAEDLTKECSSAFEKV<br>GTCLSYATGKAAMPSKECCGSVTEIKNSEPVCLCYIIQQTHN<br>GSNQIKSMGIQEGKLLQLPTTCKLTNASVSYCPKLLNIPAGS<br>PDASIFTNTNATSTTPQGPLTTTTTGGDNIGSKHSPNLAGLVA<br>VTVAVLLCSFPTEIVSAFYT
AcXYLP22	Achrdv1x17g183400	MEGFKISPSLIAISAMILVISVISVDGQISTACTNSMINSFTPCL<br>NFITGSSGNGASPTAGCCDSFKSLMSGSMDCVCLIVTGNVP<br>LSLPINRTLAISLPRACKSSVPIQCKASGVPLPAPGPVLFAPPI<br>APTADSPFSPRASKAAASPPSPSPEAFSQITPAAPPVDAAAP<br>TTNPGIRPVVTPPSASNPSYIPSPFILLVFMAILFF
AcXYLP23	Achrdv1x17g183410	MAVKGVEMVLVLVLVVVLWNGVAAQSGCTSLLMGLSPCLN<br>FVNGNSSTPSSSCCSQLNGVVQSQPRCLCSLLNGGGSSFG<br>ITINQTLALALPGACKVQTPAISQCNAAASGPAASATIPASAP<br>VGSPADFTPGGTSNETPEIPTTPSESDIPSAGAGSKTFPSTD<br>GASSGGSTIKSPLHIAAFLLFIISCASTATGF
AcXYLP24	Achrdv1x24g268370	MAAFQPSLSFLPTLAIVLAVVLFPVHAQIRAPCTASMISTFSP<br>CMNYLTNSTANGTSPTSDCCNSLKSLMSNGTDCMCLIATGS<br>IPFQIPINRTLAISLPRACNMPGVPLQCKASTAPIPAPGPIALG<br>PSLSPSAPAALTPTAPPEADATPALTPPSTTVGSEAPTSNSG<br>RPVVTPSAAEPSLGFSPSLLLTLFGAVVLKYY
AcXYLP25	Achrdv1x11g123850	MRFTMCCGIGMLQVVVLIMLAVLVADQGRADHQADTSCVNR<br>LLPCLNYLNGTHDPPNSCCDPLKSVIKSNPECLCSMISIKGT<br>KQAEEAGINVTEAQQLPGRCGQHVNPIACLTNAASPANSKN<br>SVPNSATTFSFSYSQSMPILTAVLSMILISTYLSV
AcXYLP26	Achrdv1x15g163330	MDFSKILALAMVAAVVLVAEGQPTPSCAEQLVPCVSFINATG<br>TPPATCCDPLKEAVTQQMQCLCNLYNTPGFLKSVGIDVNQ<br>ALLLPGRCNIAGGDLSSCVKASAPSSPSTEPPPATPGNGAG<br>RFSWIGMPSMFLFWAFMMLC

AcXYLP27	Achrdv1x14g154760	MGCTKFSVVAAVVVAAVLMVVAEGQSSTPS**CGQKLIPCANY INATNPPSSCCEPLREAVTKEITCLCNLYNTPGLLASFGINIT QALLLPGRCKISGDLNSC**VKASAPTSPSSSEPPPATPGNDK NGVDRIAGTGISSLLLFWASLMLY
AcXYLP28	Achrdv1x16g174090	MFFFFRKGKRMDLKMSVPLFLVIMCSWPCLGNSEDVSGTG MGMGMDGGGDESSNSLP**CLQKLMPCQPYLQSGGSGSGA PPASCCVPLKEMISGDTACLCGIFNNVDLLKTFNLTQDQAL KLPKACSANADISVC**KTDSAAPSGSPAIPTPATPSNSTTSPS NTTSPKSESAHLKNASIGGFSLIILFILNLIL
