# Supplementary figures and images for: Identification, Characterization and Expression Profiles of Xylogen-like Gene Family in Kiwifruit in Different Developmental Tissues and Under Various Abiotic Stresses
Source: Biology (Basel). 2026 Jan 31;15(3):264. doi: 10.3390/biology15030264 (PMC12896894; doi:10.3390/biology15030264)

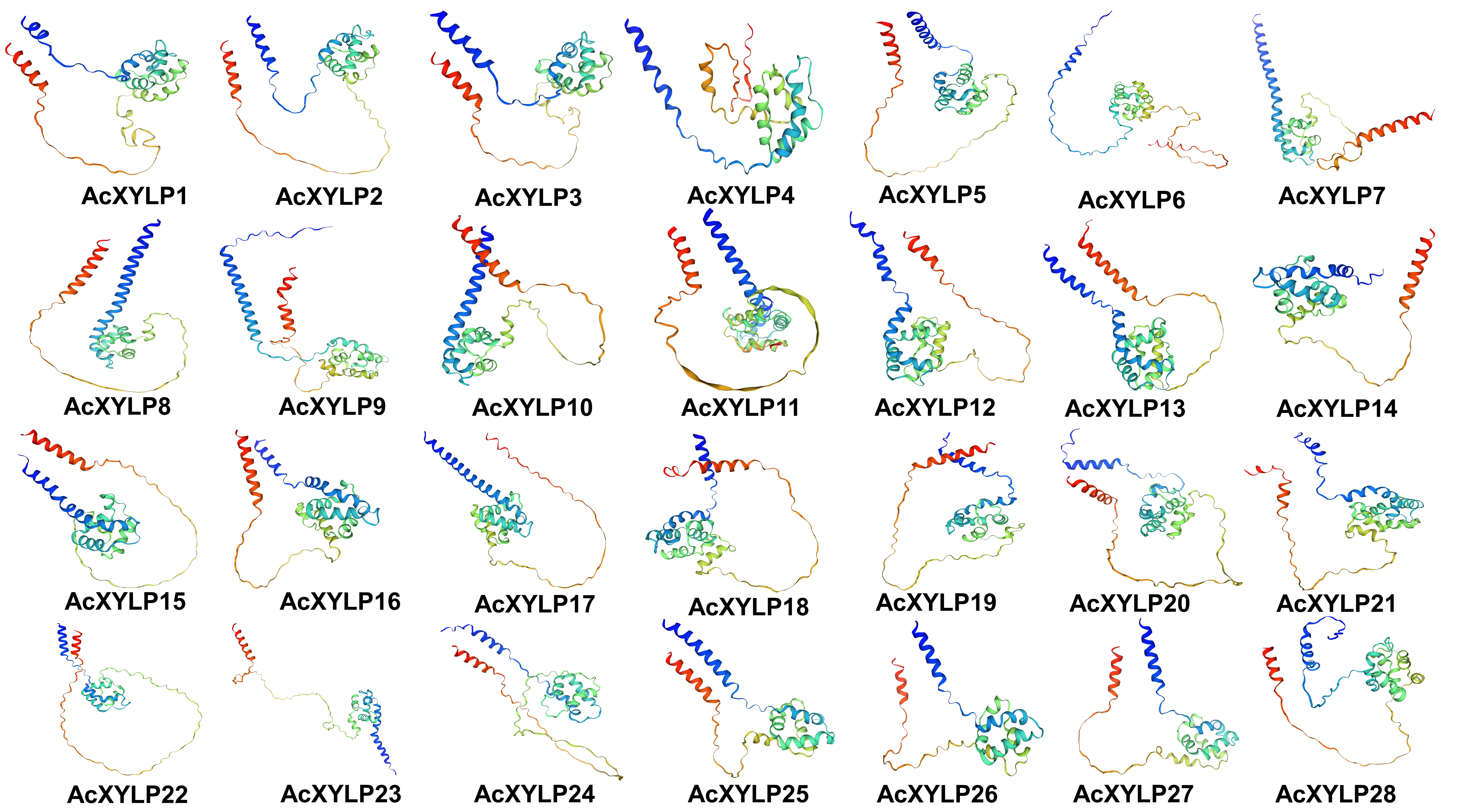

Supplement: Supplementary file 1 [file biology-15-00264-s001.zip › Fig. S2 Protein structures of AcXYLPs.tif]

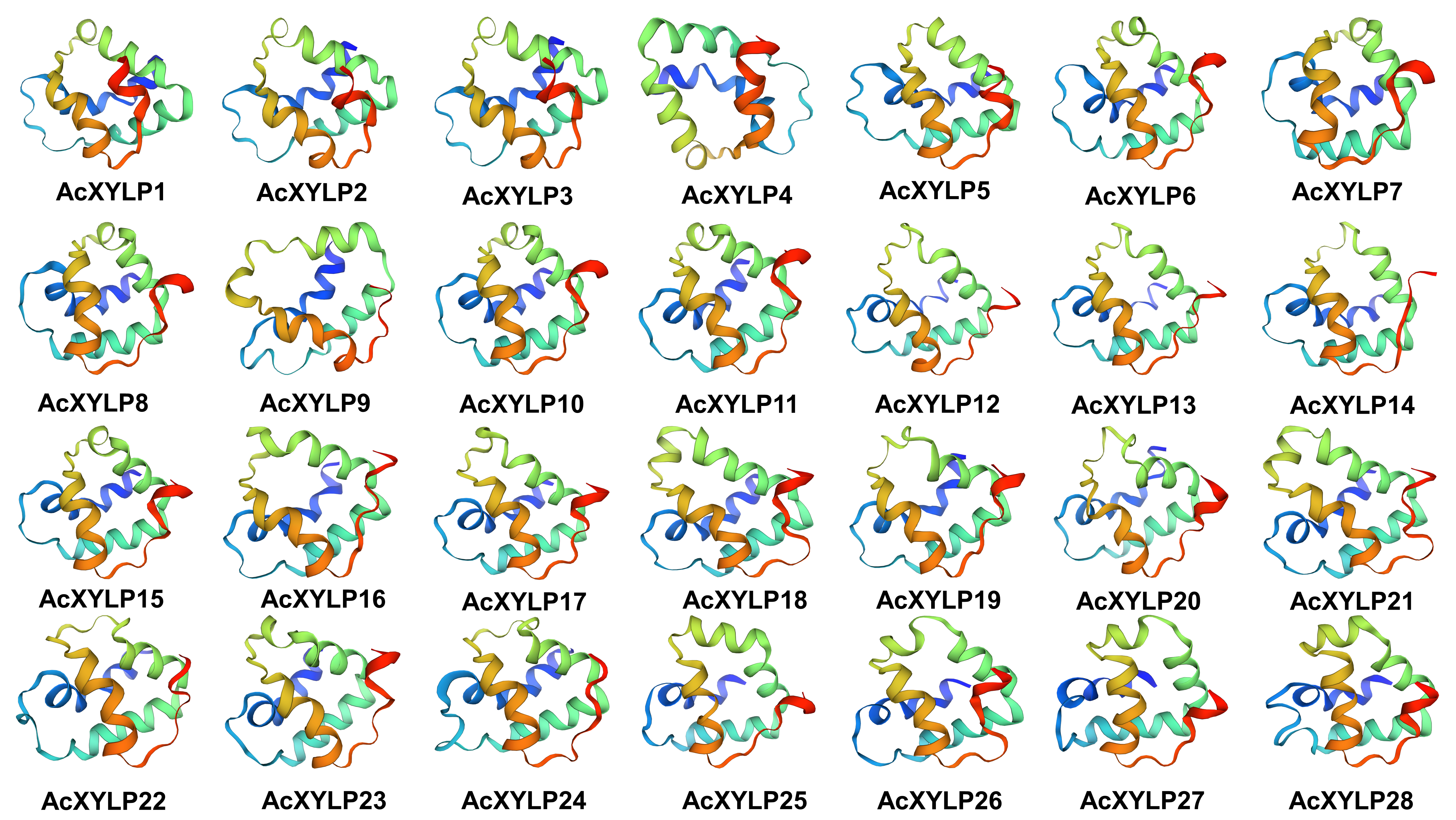

Supplement: Supplementary file 1 [file biology-15-00264-s001.zip › Fig. S3 Protein structures of conserved nsLTP domains.TIF]

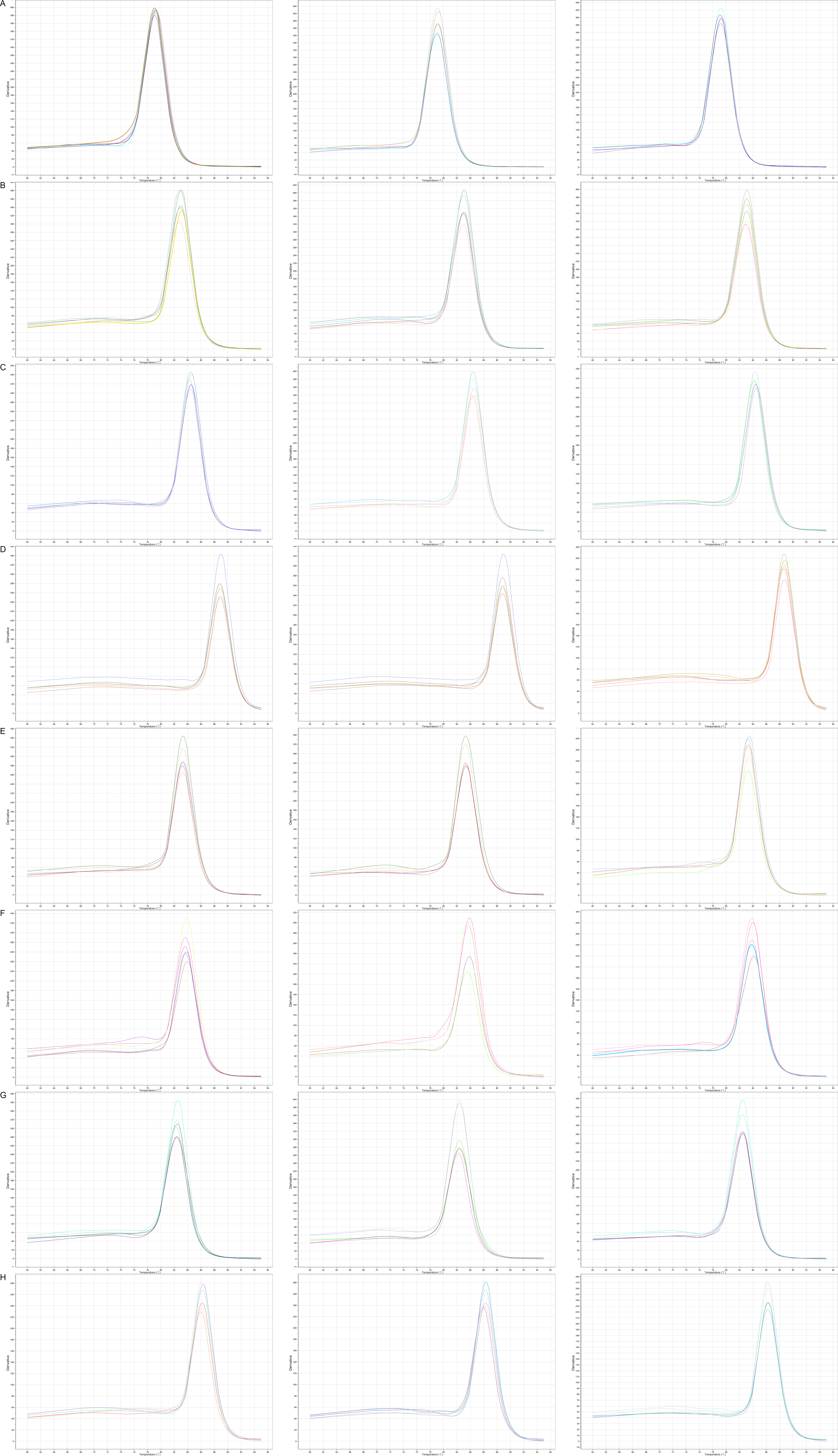

Supplement: Supplementary file 1 [file biology-15-00264-s001.zip › Fig. S4 Melting curves for qRT-PCR amplification.tif]
